# Novel digital approaches to the assessment of problematic opioid use

**DOI:** 10.1186/s13040-022-00301-1

**Published:** 2022-07-15

**Authors:** Philip J. Freda, Henry R. Kranzler, Jason H. Moore

**Affiliations:** 1 Cedars-Sinai Medical Center, Department of Computational Biomedicine, 700 N. San Vicente Blvd., Pacific Design Center Suite G540, West Hollywood, CA 90069 USA; 2 University of Pennsylvania, Center for Studies of Addiction, 3535 Market St., Suite 500 and Crescenz VAMC, 3800 Woodland Ave., Philadelphia, PA 19104 USA

**Keywords:** OUD, Opioid, Risk assessment, PRS, Machine learning, Artificial intelligence

## Abstract

The opioid epidemic continues to contribute to loss of life through overdose and significant social and economic burdens. Many individuals who develop problematic opioid use (POU) do so after being exposed to prescribed opioid analgesics. Therefore, it is important to accurately identify and classify risk factors for POU. In this review, we discuss the etiology of POU and highlight novel approaches to identifying its risk factors. These approaches include the application of polygenic risk scores (PRS) and diverse machine learning (ML) algorithms used in tandem with data from electronic health records (EHR), clinical notes, patient demographics, and digital footprints. The implementation and synergy of these types of data and approaches can greatly assist in reducing the incidence of POU and opioid-related mortality by increasing the knowledge base of patient-related risk factors, which can help to improve prescribing practices for opioid analgesics.

## Background

The prevalence and consequences of problematic opioid use (POU) continue to be serious societal issues. Of the 71,000 overdose deaths that occurred in the United States in 2019, over 70% involved opioids [1, 2]. Although the majority of fatal opioid overdoses involve illicitly manufactured and/or obtained opioids [2], like fentanyl and heroin, most individuals who misuse opioids are initially prescribed opioids for pain management [3–5]. Therefore, developing effective approaches to identify risk factors associated with the initiation of POU in healthcare settings can contribute to safer opioid prescribing practices and fewer deleterious consequences of prolonged and elevated opioid use.

Major challenges associated with identifying the risk factors of POU stem, in part, from the complexity of the concept itself. POU is described by many terms, each existing on a continuum of severity with opioid misuse considered the least severe and opioid dependence commonly considered the most severe [6]. However, this varies in the literature [7]. In addition to these terms, there also exists a Diagnostic and Statistical Manual of Mental Disorders, Fifth Edition (DSM-5) clinical diagnosis for opioid use disorder (OUD), which incorporates terms from the POU continuum and provides a severity scale based on the number of criteria met [8]. Although the OUD diagnosis subsumes the POU continuum, terms like misuse, abuse, addiction, and dependence are still commonly used, both in the literature and in patient medical records, and can describe POU with or without an accompanying OUD diagnosis [9–11]. Because of the terminologic ambiguity and potential overlap among terms, the reported prevalence of POU-related traits varies widely [7, 9, 10] and is commonly not reported or underreported [12–15]. However, despite difficulties arising from the challenges of describing and quantifying POU as a determinant of health, many individuals exposed to opioid analgesics develop some level of problematic use.

**Fig. 1:**
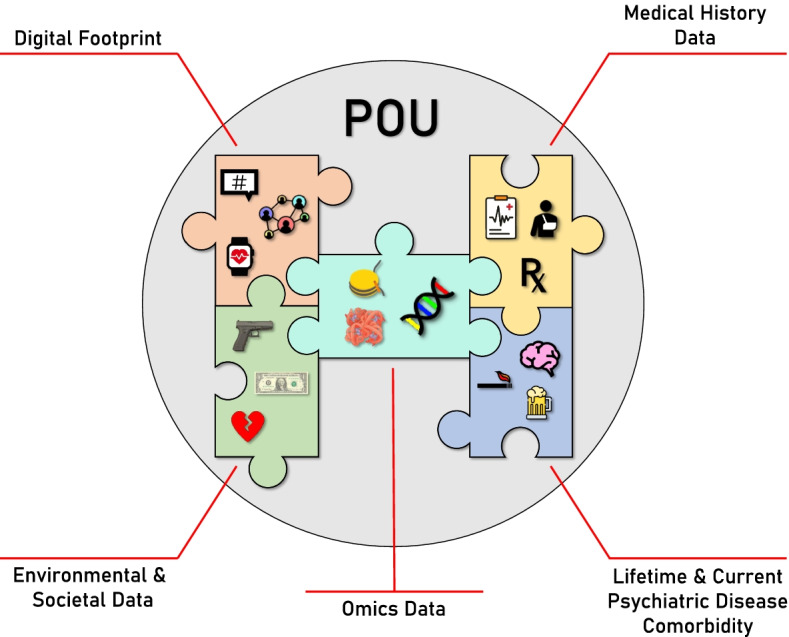
Conceptual image illustrating potential sources of POU risk using puzzle pieces. The right puzzle pieces (Medical History Data and Lifetime & Current Psychiatric Disease Comorbidity) represent phenotypes that are usually mined from electronic health records (EHR), clinical notes, or structured assessments/questionnaires. The left puzzle pieces (Digital Footprint and Environmental & Societal Data) represent mostly environmental data that can be obtained from EHR, clinical notes, structured assessments/questionnaires, social media, and biometrics. The middle puzzle piece (Omics Data) represents large-scale genetic, epigenetic, proteomic, and metabolomic data. This piece links the two sides of the image as there are likely synergistic and/or causal relationships with the environment and the greater phenotype

A second difficulty in identifying risk factors for POU results from the sources of risk themselves. Variation in the POU phenotype comes from three sources: genetic variation, environmental variation, and the interaction between the two [16], the first two of which have been shown to be significant factors underlying POU prevalence and severity [17–27]. However, although humans live complex lives where important interactions and events occur regularly, potential and perhaps significant sources of risk are commonly overlooked or not recognized [28–30]. Expansion of the genetic and environmental dimensions of risk yields many sources to be considered including omics data, electronic medical records, demographics and personal histories, and digital footprints, which include, but are not limited to, social media activity (Fig. 1). Thus, capturing the greater phenotypic and environmental profile of an individual has the potential to greatly improve risk assessments for POU.

The complexity of POU as a measurable trait presents many challenges from a data science perspective due to ambiguous POU-related terms and their complex, but poorly explored, root causes [6, 7, 28, 30, 31]. These issues make powerful digital approaches difficult to implement despite many recent advances in the fields of artificial intelligence, bioinformatics, and computational biomedicine. In this review, we discuss the importance of considering all available sources of data when assessing disease risk, ways in which POU can be explored as a trait of interest in biomedical research, and novel digital approaches and technologies that can be utilized to explore complex and diverse datasets. Our goal is to illustrate how diversifying and expanding both data acquisition and methodology can improve POU risk assessment and prediction, potentially alleviating adverse impacts of POU on patients, families, and society.

## Review methods

**Fig. 2:**
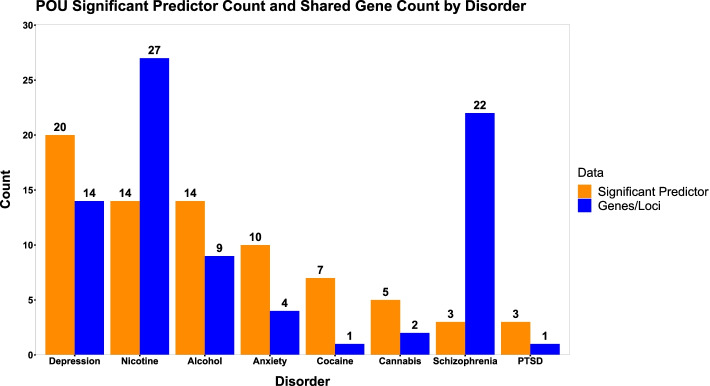
Bar plot (orange) of the count of psychiatric disorders and substance use disorders that were significant indicators of problematic opioid use (POU) phenotypes in our literature search (see Review Methods for criteria) and bar plot (blue) of the shared gene/locus count between psychiatric disorders and substance use disorders with POU (see reference [28] for methodology). Shared gene/locus associations reflect the relative representation of each disorder as significant predictors of POU. Depression, nicotine and alcohol use disorders, and anxiety disorders show high shared genetic liabilities with POU and are the most significant indicators of POU. However, schizophrenia displays high shared genetic liability with POU despite lower POU prediction

For compiling the significant predictors of POU in the literature, we used the search terms “risk factors of opioid use disorder” and “predictors of opioid use disorder” in Google Scholar and identified scientific research articles and clinical studies over the past 10 years within the first 100 search results of both search terms. We used this information to create Fig. 2 (orange bars). To include the gene/locus information presented in Fig. 2 (blue bars), we integrated data from a literature search protocol implemented in a previous review [31].

## Risk factors of POU

Risk factors of POU are likely vast and include sources of data that may not be obvious or readily available. However, risk factors can stem from five basic sources (Fig. 1): Lifetime and current psychiatric disease comorbidities that include both mental disorders and substance use disorders (including previous opioid use) [32–34], medical histories from EHR data and clinical notes [35, 36], environmental and societal factors that include demographics and personal histories [28], digital footprints including social media and biometrics [37–39], and omics data which comprise genetic, epigenetic, transcriptomic, and other large-scale biological data [19–27]. In Fig. 1, the right side of the puzzle image represents medical/biological phenotypes while the left side represents mostly environmental features. In the center, omics data links the two sides of the conceptual image, illustrating that a patient’s genotype likely has synergistic and/or causal relationships with the environment and the greater phenotype.

Of the above sources of information, comorbid lifetime and current psychiatric disease diagnoses are perhaps the most significant indicators of POU and its development. For example, tobacco use disorder (TUD) has a comorbidity rate as high as 98% in populations of patients in medication-assisted treatment programs for POU [40–43]. TUD is also a common pre-morbid risk factor of POU, associated with the initiation and persistence of opioid use and OUD development [44]. Similar relationships with POU have also been described for cocaine use disorder [45, 46], alcohol use disorder [47], and cannabis use [48]. Mood and anxiety disorders are also commonly associated with POU. Depression alone has been linked to the risk of opioid relapse [34], opioid misuse [49], a diagnosis of OUD [50], and risk of death from OUD-related overdose [51], while anxiety disorders have been linked to opioid relapse [34], non-medical use [52], and misuse [53]. Figure 2 (orange bars) illustrates how often a psychiatric disease was found to be a significant indicator of a POU phenotype within our literature search criteria (see Review Methods) [10, 17, 44, 47, 49, 50, 52–81]. Depression, nicotine use/smoking status, alcohol use, and anxiety disorders were the most common predictors of POU (Fig. 2; orange bars). In addition to these comorbidities as risk factors of POU, the strongest indicators of future POU development are past instances of opioid use or POU [10, 17, 44, 47, 49, 50, 52–81]. Due to the nature of the relationship between psychiatric disease and POU, histories of substance use/abuse and mental illness, although sometimes challenging to obtain, are important sources of data for POU risk assessment.

Other major contributions to POU risk are an individual’s genetic and epigenetic profiles [22, 24, 27, 82, 83]. Several genetic variants have been associated with opioid use and dependence via genome-wide association studies (GWAS) and candidate gene studies. Foremost of these are variants located in the gene encoding the μ-opioid receptor 1 (*OPRM1*), which have putative roles in the genetics and pharmacogenetics of POU [22, 24, 27, 82]. Structural and epigenetic variation has also been associated with POU. As examples, copy number variation in the genes *KCND2* and *MAP3K4* have been associated with opioid dependence [83] and opioid exposure has been shown to induce marked changes in histone acetylation, histone methylation, DNA methylation, and non-coding RNA expression, which collectively have the capacity to affect the expression of many gene targets [84]. Many of the genetic variants associated with various POU phenotypes have also been associated with other psychiatric disorders. Figure 2 (blue bars) illustrates how many genes or loci, per disorder, have also been associated with a POU phenotype [31]. Somewhat reflecting the relative representation of each disorder as important predictors of POU in the literature (Fig. 2; orange bars), genes associated with nicotine use, schizophrenia, depression, and alcohol use have the strongest shared genetic liabilities with POU (Fig. 2; blue bars) [31]. Although considerable research has been conducted to identify genetic factors contributing to POU and those shared with other disorders, new variants are being discovered using modern and robust approaches, highlighting the importance of gathering high quality genomic data when assessing POU risk [85].

A patient’s medical history, usually accessible via EHR structured data and clinical notes, can provide extensive information useful for POU risk assessment. However, the structure and format of EHR data and clinical notes are not uniform and can be limited and/or difficult to navigate depending on the healthcare institution [86, 87]. Despite this, efforts should be taken to collect as much data as possible on each patient when considering risk of POU as many the non-biological predictors associated with it include level of education, marital status, income, geographic location, and insurance status [17, 49, 63, 88]. There are also, of course, biological predictors of POU risk that include but are not limited to BMI, race, age, sex, medication history, procedure/operation history, comorbid disorders, and endophenotypes [17, 49–51, 88, 89]. Endophenotypes are defined as physiological traits related or contributing to a disease trait [89]. For example, an endophenotype for hypertension is blood pressure. Clinical notes can also be a robust source of risk indicators for POU as they can capture critical pieces of information not available in structured EHR data. During a clinical encounter, a patient may discuss topics with a healthcare professional that may be associated with risk of POU development but not be captured in specific EHR data entry fields [36]. If this information is recorded electronically, natural language processing (NLP) approaches can identify features from typed language that can assist in the development of risk assessment protocols [64, 90]. Other potential sources of risk can be derived from pain and mental health assessments during hospital stays as ratings of both have been associated with POU [66, 91, 92]. An example of a system designed to capture these types of information is the Patient-Reported Outcomes Measurement Information System® (PROMIS®), which produces scores for several metrics including anger, anxiety, depression, pain behavior, and pain interference. If available, these types of data should be incorporated in POU risk assessments due to the strength of the associations between mental health and perceived pain with POU.

Perhaps the most elusive source of data for POU risk assessment comes from an individual’s environment, both physical and digital. As highlighted above, many aspects of an individual’s personal life have been shown to be significant indicators of POU [17, 18]. Much of this information is not readily available in EHR data but may be accessible via clinical notes, structured assessments, or questionnaires or derived from social media data. For example, text analyzed from opioid-related groups on Reddit.com identified significant risk factors for OUD, opioid relapse, and recovery seeking behavior [39]. However, most striking is that 72% of individuals who had relapsed exhibited strong emotional language in 2 of 10 possible emotional categories – “Joy” and “Negative”. This implies that relapse is related to extreme emotions and treatments aimed at supporting the regulation of emotion could reduce the risk of relapse or increased opioid use [39]. Another example comes from utilizing over 9000 opioid-related posts and demographic information from Twitter [37]. Using NLP and ML classification, a significant correlation was found between posts classified as related to opioid misuse and real-time overdose deaths rates in Pennsylvania [37]. Although powerful approaches, these examples are either anonymous or not linked to a person’s medical history and therefore difficult to use in POU risk assessment in clinical settings. However, text and demographic data from Facebook, linked to an individual’s EHR data, was implemented in various ML algorithms to predict medical conditions That include diabetes, hypertension, depression, and digestive issues [38]. Further, the inclusion of data from Facebook significantly improved the prediction accuracy of models in 18 of the 21 disease categories explored [38]. Notably, among mental health conditions, predictions of anxiety, depression, and psychosis showed the most improvement [38]. Data from social media may also provide additional insight into risk factors, which EHR data cannot, insofar as many aspects of patients’ personal histories and daily experiences are not available in the medical record. These studies illustrate how encoded social media language and demographic data, much like genetic variants, can be used as disease factors in ML approaches. Also, the predictive implications for psychiatric traits suggest that social media data could be powerful in assessing factors contributing to the risk of many substance use disorders including POU [38]. Finally, biometric data from sources like smart watches and phone apps can provide insight into a patient’s exercise regime or sleep schedule, among other things. Because greater physical activity has been associated with lower opioid use [17] and greater opioid use has been associated with interrupted sleep [93], biometric data can be useful in POU risk assessment. Linking these types of data to a patient’s medical history can significantly improve POU risk assessment.

## Quantifying and categorizing POU

Because POU is a broad phenotype, categorizing it or quantifying its levels as a phenotype or response variable presents challenges. Terms like use, misuse, dependence, addiction, and abuse do not have universally accepted criteria and, therefore; commonly do not translate into useful comparisons across experiments [7]. However, in the case of OUD, because it is a clinical diagnosis, both its presence or absence or its severity can be useful phenotypes for risk assessment. Despite this, there are limitations in relying on the diagnosis of OUD for risk assessment. The first of these is the underdiagnosis of the disorder, which results in a failure to detect risk factors and/or underperformance of predictive models [12–15]. The second limitation is that there is variation in the interpretation of criteria and/or severity of OUD diagnoses across clinicians or healthcare settings [94]. A possible solution to this limitation is the quantification of opioid exposure or usage. Although there are many opioid-based analgesics prescribed in clinical settings, the dosage of various opioids can be standardized as morphine equivalent dosage (MED), also called morphine milligram equivalent daily dosage (MEDD) or morphine milligram equivalents (MME) [95]. Other conversion metrics that exist internationally include the defined daily dose (DDD) [96] and oral morphine equivalent (OMEQ) [97]. A potential downside of this approach is that MME does not account for biological, genetic, or pharmacokinetic differences among individuals, highlighting the importance of collecting data from diverse sources when performing risk assessments and using MME in combination with a clinical diagnosis of OUD or the presence of other POU-related terms, as may be available.

MME is also advantageous over an OUD diagnosis or POU-related terms, particularly those derived from the EHR, in that it is a continuous variable. Continuous variables are statistically more powerful in regression-based approaches in ML pipelines and can be converted to discrete variables to take advantage of classification-based approaches. Discrete variables, like OUD presence/absence or severity as measured by criteria met, are limited only to classification-based approaches as they cannot be inherently converted to continuous variables. MME can be converted into discrete levels based on pre-determined dosage ranges or by increments of change before and after a medical procedure like surgery.

Predicting MME after surgery can be a powerful indicator of surgical success as the primary goal of elective surgery is to decrease patient pain, and therefore MME. However, many patients’ experience greater pain after surgery [98], potentially leading to higher levels of MME. Identifying the contributors to this type of outcome can assist clinicians in determining whether surgery is in the best interest of the patient, as high levels of MME are associated with death by opioid overdose and opioid-related toxicity [81]. With increasing awareness of this risk, efforts are being made to reduce the use of opioid analgesics by using, for example, non-steroidal anti-inflammatory drugs or other non-opioid analgesics when surgery is indicated [99, 100]. In addition to MME, it is also important to collect and include pain ratings provided by the patient, both before and after surgery [92]. These data can be merged with MME to predict the pain response following surgery, as MME and pain are positively correlated [98].

## Advances and limitations of digital approaches for POU risk assessment

### Polygenic risk scores

Polygenic risk scores (PRSs) are a useful method of estimating an individual’s genetic risk for a specific trait and a promising approach for disease risk assessment. The literature on PRSs for POU, although limited, is growing. In a recent study of a large, mixed ancestry cohort, PRSs were calculated for four substance use traits (alcohol use disorder, OUD, smoking initiation, and lifetime cannabis use) [101]. Among African Americans, the PRS for alcohol use disorder and among European Americans the PRS for alcohol use disorder, OUD, and smoking initiation were associated with their respective Diagnostic and Statistical Manual of Mental Disorders (DSM) diagnoses and criterion counts – highlighting the predictive power of PRS. Phenome-wide association studies (PheWAS) of these PRSs showed the most associations with other substance use phenotypes. For example, the PRS for OUD was associated with 7 substance-use phenotype categories, the strongest of which was the DSM-5 diagnosis of tobacco dependence. A large, meta-GWAS of European Americans identified loci for problematic alcohol use with significant genetic correlations between problematic alcohol use and 138 phenotypes [102]. The highest genetic correlations were with other alcohol phenotypes, tobacco phenotypes, and psychiatric disorders including depression, schizophrenia, and bipolar disorder. These results highlight the utility of PRS for identifying substance use disorder risk and shared genetic liabilities among various psychiatric comorbidities.

Despite the potential utility of PRS for identifying and quantifying disease risk, it has inherent limitations. Many studies are of samples with a specific ancestral background, which limits the applicability of the PRS. Despite this, limiting cohorts to a specific ancestral background is a common practice as to include multiple ancestral groups, except by meta-analysis, overlooks their different genetic architectures and allele frequencies [103]. PRSs have been shown to provide only limited utility across population groups [104]. Further, PRSs can differ significantly even within population groups when the data are stratified by characteristics such as socioeconomic status, age, and sex [105].

Due to the difficulty in recruiting and assessing large samples for GWAS, it is becoming increasingly common to use EHR data linked to genomic data for PRS prediction due to the wide array of phenotypes available, the speed at which studies can be performed, and the potentially high levels of reproducibility. However, these benefits come with their own challenges. A recent review highlights some of the challenges of linking medical records to genomic biobank data and considerations on how to limit or remove them [89]. Potential difficulties include properly defining disease phenotypes universally (e.g., which for POU can be difficult given the absence of a universally agreed-upon phenotype), complexities and redundancies in the International Classification of Diseases (ICD) code systems, the limited applicability of GWAS summary statistics using datasets that represent only one ancestral group, and the small effect sizes associated with common variants [89]. However, these limitations and challenges can be mitigated by utilizing the right tools. For instance, much of the ambiguity introduced by ICD codes or complex disease phenotypes, like POU, can be alleviated by using phenotyping algorithms specifically designed to deal with diverse types of data, like EHR data [89, 106–108]. The Phenotype KnowledgeBase is a public repository that houses numerous algorithms for this distinct purpose and can help to identify difficult phenotypes [109]. Furthermore, by utilizing ML-based approaches, EHRs can drastically improve PRS study design, reproducibility, and prediction as the use of EHR-mined phenotypes can reduce the time required to build a cohort while ensuring that the population in which the PRS is being estimated is representative of the healthcare system population, increasing overall diversity [89].

Another limitation of PRSs is that they do have the statistical power to detect the existence of epistatic (i.e., gene-gene) interactions when assessing polygenic risk. A recent approach, the Multilocus Risk Score (MRS), uses model-based multifactor dimensionality reduction to detect epistasis between loci. A study that tested the efficacy of this approach compared standard PRS methods with the MRS method in a diverse collection of simulated datasets [110]. In 335 of 450 datasets, MRS produced greater area under the receiver operating characteristics (auROC) curve than PRS, even when no epistatic interactions were detected. Using a Wilcoxon signed rank test, the improvement of MRS over PRS was significant (*P* < 10^− 5^) [110]. Thoughtful considerations and improvements, as highlighted in [110], can be used to improve the efficacy of PRS so that genetic relationships for POU and other disorders can more robustly be described, detected, and generalized.

### Machine learning and artificial intelligence

Advances in ML are appearing daily and several of these have the potential to be useful in OUD research. There has been substantial attention given to neural networks as a ML method. In particular, deep learning (DL) has been developed to extend the architecture of neural networks to include many layers of nodes, thus greatly improving their ability to perform tasks such as image recognition [111]. It will be important to explore how best to adapt these algorithms to the study of OUD. One promising approach is the application of knowledge of biology and biochemical pathways to guide the architecture of a DL neural network [112]. Adapting this approach to the conduct of research on OUD is promising because researchers can build on the existing knowledgebase to help reduce the computational complexity of algorithms by reducing the feature spaces in informative ways. Another promising area to explore is automated ML (autoML). One of the challenges of ML is knowing which methods to select. Each method looks at the data in a different way and it is difficult to know a priori which method is best for detecting unknown patterns in a specific data set. The goal of auto ML is to let the computer explore the space of possible algorithms and parameter settings to automatically select the best method [113]. An example of an autoML package that can be used for big biomedical datasets is TPOT which uses genetic programming (GP) to optimize potential ML pipelines [114–116]. The goal of the GP applied in TPOT is to assign fitness scores to each ML pipeline and through generations of reproduction and mutation, arrive at an optimized solution in terms of model accuracy. Approaches like this can take some of the guesswork out of ML, as the technology becomes more accessible to individuals with less experience or skill in applying the methods. Finally, interpretation is key to translating ML results into improvements in our understanding of a phenomenon or in leading to new biological or clinical studies. Making sense of ML results is, in some cases, more challenging than developing the models themselves. This is where the human element comes in. ML and artificial intelligence (AI) are tools that need human interpretation and experience to turn data into knowledge. Interpretability, transparency, and trust are new frontiers in ML research.

In their application to POU, ML algorithms and approaches aimed at extracting phenotypes from EHR data are extremely useful because of the number of terms, diagnoses, and metrics that can translate to some level of problematic use. Several recent studies have incorporated and evaluated a diverse set of ML methods to derive phenotypes from EHR data for disorders including atopic dermatitis [117], rheumatoid arthritis [118, 119], and type 2 diabetes mellitus [120]. In the case of type 2 diabetes, several ML methods were evaluated including k-nearest neighbor, decision tree, random forest, support vector machine, and naïve Bayes. All of these approaches yielded higher auROC (average across methods was 0.98) than the state-of-the-art linear regression algorithm (auROC = 0.71) [120]. ML algorithms, in addition to detecting occurrences of a particular phenotype, can also enrich current phenotypes by expanding them into levels of severity or subtypes. As examples, two recent papers used latent class analysis to identify sub-phenotypes of acute respiratory distress syndrome [121] and pediatric sepsis [122]. Elucidating phenotype stratification is important as different sub-phenotypes often require different treatment strategies and responses. POU could particularly benefit from EHR mining as the phenotype is diverse and complicated and improvements in its detection will improve both treatment and risk assessment strategies as the knowledge base expands.

Although NLP is a branch of AI, its usefulness and robustness in the identification and risk assessment of POU warrants a focused discussion. ICD codes, which are used by physicians to diagnose and categorize patients, can help to identify both POU and OUD. However, standardized systems like ICD codes or EHR fields often underestimate the total of number of patients who exhibit one or more of these diagnoses [123]. When used in clinical settings, NLP creates a dictionary of terms and phrases from text sources (structured or unstructured) using automated algorithms to identify individuals who have, or may be at risk of having, a diagnosis of interest. Thus, NLP may identify patterns from clinical notes associated with a certain diagnosis that standardized classifications (e.g., ICD codes) cannot. Indeed NLP-assisted manual review of EHR data has been shown to greatly assist the classification of POU by identifying additional instances of POU that ICD code identification alone misses [90, 124]. However, in these examples, NLP methods alone did not identify all patients with POU ICD codes. This lack of overlap highlights the importance of using both detection methods in tandem to enhance POU identification. NLP also has the potential to identify risk factors of POU. For example, NLP methods accurately predicted opioid agreement violations in chronic non-cancer pain patients (sensitivity of 96.1%, specificity of 92.8%, and positive predictive value of 92.6%) [125]. Because of the high probability of developing OUD when prescribed opioids, clinicians and patients can enter into an opioid or pain management agreement in which the patient agrees to undergo random drug screenings and/or pill counts. Identifying patients that have violated or are at risk of violating these agreements is important to responsible opioid dispensing. Finally, improvements in text-based classifiers can have significant positive effects on NLP performance. A recent study highlights one such improvement. The researchers performed manual reviews of hospital discharge summaries and identified several text classes describing potential POU [36]. Annotated sentences were used to generate features using the open-source knowledge bases Empath [126], the Unified Medical Language System [127], and PyConText [128]. Several ML classifiers were used to predict sentence classification. Of these classifiers, AutoGluon had the best performance among classes in testing sets (average *P* = 81.4, *R* = 77.8, and *F*_*1*_ = 78.2 compared to average *P* = 81.2, *R* = 65.8, and *F*_*1*_ = 70 in logistic regression). AutoGluon is an autoML package that incorporates DL for text, image, and tabular data classification from structured data and focuses on multi-layer model stacking instead of model and hyperparameter selection [129]. The stacking allows basal models predictions to improve future models using both prediction information and feature space from the previous layer. This yields greater accuracy and faster computational times than several other autoML frameworks [129]. AutoML packages like TPOT and AutoGluon represent significant advances in model selection and optimization and have the potential to improve significantly the classification and prediction of complex phenotypes like POU.

## Conclusions and synthesis

**Fig. 3:**
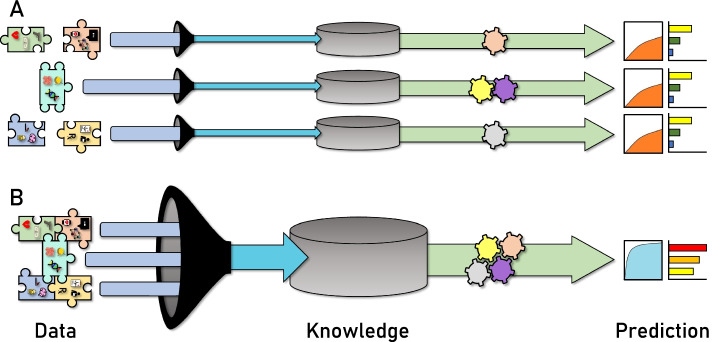
Conceptual Image illustrating the flow from data to knowledge to prediction. In **A**, the puzzle pieces from Fig. 1 are not connected, illustrating that each part of the puzzle is being treated separately. Three individual data extraction methods are shown with blue arrows passing through a filter. The filter represents feature cleanup and engineering. Data build a knowledge base (gray cylinder) before various methodologies (gears) create models for prediction. The area under the receiver operating characteristic curve (auROC) plots and feature importance plots (horizontal bar graphs) represent levels of model accuracy. In **B**, the puzzle pieces from Fig. 1 are connected. Individual data streams are filtered together to create a single source of data that contributes to a larger knowledge base. Multiple methodologies working together (interlinked gears) result in better models (as measured by higher auROC and greater feature importance)

In this review we have highlighted the difficulties in classifying and identifying POU as a biomedical phenotype, the complex and potential risk factors associated with POU to inform feature identification and engineering, recommendations on how to quantify and classify the phenotype itself, and several methods, approaches, and advancements in the fields of ML, AI, and bioinformatics to identify POU and its risk factors. Throughout the review, we have sought to emphasize the importance of incorporating diverse and varied types of data and multiple methods and approaches to assess and predict POU risk. Figure 3 conceptually reinforces this idea. Each pipeline alone has its own potential to yield important features and risk predictions. However, combining various sources of data and methodological pipelines increases the potential knowledge base, which yields more robust models and better identification and prediction. The workflow from data to knowledge to prediction can be greatly improved by accessing all available data sources and incorporating novel digital approaches. It is our recommendation that future work in the fields of POU prediction and POU risk assessment incorporates diverse types of data (e.g., environmental data, digital footprints, comorbidities, and omics data) as well as multiple methodologies to create robust models and pipelines. Although the collection of varied data can be particularly challenging, we implore researchers to develop novel ways to capture the complex lives of their cohort(s). It is our hope that improving the knowledge base of POU will lead to the development of more efficient and accurate opioid risk prediction/assessment techniques, which is essential to limiting the exposure of individuals at risk and managing this public health crisis.

## Abbreviations

AI: Artificial intelligence
autoML: Automated machine learning
auROC: Area under the receiver operating characteristics curve
DL: Deep learning
DSM: Diagnostic and statistical manual of mental disorders
DSM-5: Diagnostic and statistical manual of mental disorders, fifth edition
EHR: Electronic health record(s)
GP: Genetic programming
GWAS: Genome-wide association study(ies)
ICD: International classification of diseases
ML: Machine learning
MME: Morphine equivalent dose
MRS: Multi-locus risk score(s)
NLP: Natural language processing
OPRD1: δ-opioid receptor 1
OPRM1: μ-opioid receptor 1
OUD: Opioid use disorder
PheWAS: Phenome-wide association study(ies)
POU: Problematic opioid use
PRS: Polygenic risk score(s)
SNP: Single nucleotide polymorphism
TUD: Tobacco use disorder
